# Multi-Method Analysis of Histopathological Image for Early Diagnosis of Oral Squamous Cell Carcinoma Using Deep Learning and Hybrid Techniques

**DOI:** 10.3390/cancers15215247

**Published:** 2023-10-31

**Authors:** Mehran Ahmad, Muhammad Abeer Irfan, Umar Sadique, Ihtisham ul Haq, Atif Jan, Muhammad Irfan Khattak, Yazeed Yasin Ghadi, Hanan Aljuaid

**Affiliations:** 1 Department of Electrical Engineering, University of Engineering and Technology, Peshawar 25000, Pakistan; mehranahmad7242@gmail.com (M.A.); atifjan@uetpeshawar.edu.pk (A.J.); m.i.khattak@uetpeshawar.edu.pk (M.I.K.); 2 AIH, Intelligent Information Processing Lab (NCAI), University of Engineering and Technology, Peshawar 25000, Pakistan; umar.sadique@uetpeshawar.edu.pk (U.S.); 16pwmct0478@uetpeshawar.edu.pk (I.u.H.); 3 Department of Computer Systems Engineering, University of Engineering and Technology, Peshawar 25000, Pakistan; abeer.irfan@uetpeshawar.edu.pk; 4 Department of Mechatronics Engineering, University of Engineering and Technology, Peshawar 25000, Pakistan; 5 Department of Computer Science, Al Ain University, Al Ain 15551, United Arab Emirates; yazeed.ghadi@aau.ac.ae; 6 Computer Sciences Department, College of Computer and Information Sciences, Princess Nourah Bint Abdulrahman University (PNU), Riyadh 11671, Saudi Arabia

**Keywords:** oral squamous cell carcinoma (OSCC), HOG, GLCM, LBP, hybrid model, PCA, SVM

## Abstract

**Simple Summary:**

The research aimed to address the challenges in the early diagnosis of Oral Squamous Cell Carcinoma (OSCC), a critical concern given its high fatality rate and global prevalence. Through the development of hybrid methodologies, the study sought to improve early diagnosis, reduce the burden on pathologists, and enhance the accuracy of OSCC diagnosis. By employing transfer learning, a combination of CNN and SVMs, and a fusion of deep and texture-based features, the research achieved a significant overall accuracy of 97.00%, effectively addressing the critical problem of timely and accurate OSCC diagnosis.

**Abstract:**

Oral cancer is a fatal disease and ranks seventh among the most common cancers throughout the whole globe. Oral cancer is a type of cancer that usually affects the head and neck. The current gold standard for diagnosis is histopathological investigation, however, the conventional approach is time-consuming and requires professional interpretation. Therefore, early diagnosis of Oral Squamous Cell Carcinoma (OSCC) is crucial for successful therapy, reducing the risk of mortality and morbidity, while improving the patient’s chances of survival. Thus, we employed several artificial intelligence techniques to aid clinicians or physicians, thereby significantly reducing the workload of pathologists. This study aimed to develop hybrid methodologies based on fused features to generate better results for early diagnosis of OSCC. This study employed three different strategies, each using five distinct models. The first strategy is transfer learning using the Xception, Inceptionv3, InceptionResNetV2, NASNetLarge, and DenseNet201 models. The second strategy involves using a pre-trained art of CNN for feature extraction coupled with a Support Vector Machine (SVM) for classification. In particular, features were extracted using various pre-trained models, namely Xception, Inceptionv3, InceptionResNetV2, NASNetLarge, and DenseNet201, and were subsequently applied to the SVM algorithm to evaluate the classification accuracy. The final strategy employs a cutting-edge hybrid feature fusion technique, utilizing an art-of-CNN model to extract the deep features of the aforementioned models. These deep features underwent dimensionality reduction through principal component analysis (PCA). Subsequently, low-dimensionality features are combined with shape, color, and texture features extracted using a gray-level co-occurrence matrix (GLCM), Histogram of Oriented Gradient (HOG), and Local Binary Pattern (LBP) methods. Hybrid feature fusion was incorporated into the SVM to enhance the classification performance. The proposed system achieved promising results for rapid diagnosis of OSCC using histological images. The accuracy, precision, sensitivity, specificity, F-1 score, and area under the curve (AUC) of the support vector machine (SVM) algorithm based on the hybrid feature fusion of DenseNet201 with GLCM, HOG, and LBP features were 97.00%, 96.77%, 90.90%, 98.92%, 93.74%, and 96.80%, respectively.

## 1. Introduction

Oral cancer is characterized by the development of abnormal cells in the oral cavity and is incurable once it has advanced to a later stage. OSCC is the most common type of oral cancer that begins in the oral cavity [1,2]. A type of cancer called oral cancer can develop in the head and neck. The term oral cancer encompasses a wide range of cancers that can occur in various parts of the mouth, including the tongue, floor of the mouth, lips, gums, hard palate, cheek lining, and jawbones. Oral cancer is a global health concern and is considered a deadly disease that ranks as the seventh most common cancer throughout the whole globe the fifth most common cancer in men and the eighth most prevalent cancer in women worldwide [3]. GLOBOCAN reported 377,713 new cases of oral cancer identified in 2020, with 264,212 of those cases were male and the remaining cases were female. In these cases, 177,758 patients died, 125,023 of whom were men, and 52,736 were women [4]. The incidence of cancer is increasing in Pakistan, and according to GLOBOCAN, oral cancer is the second most common type of cancer in Pakistan [5]. According to data from the Global Cancer Research Fund International, each year, there are approximately 24597 new cases of oral cancer recorded in Pakistan, 17,473 of which are males and the remaining 7124 are women. There are also about 15,127 deaths due to this fatal disease, with 10,620 of them being men and 4507 being women [3]. The majority of oral cancer cases occur in countries with low to moderate incomes; in particular, Southeast and South Asia account for approximately two-thirds of the cases [6]. Tobacco, betel quid, alcohol, poor oral hygiene, Human Papillomavirus (HPV) infection, ethnicity, geographic location, and family history are factors associated with the development of OSCC. Most OSCCs develop from oral potentially malignant diseases (OPMDs), such as oral lichen planus, Erythroplakia, Erythroleukoplakia, and Leukoplakia, with a 1% chance of becoming cancerous [7]. Oral cancer has a high death rate as it is usually diagnosed in later stages; hence, recovery or treatment is almost impossible. The early stages of OPMDs and OSCC are frequently symptomless and may appear as innocuous lesions, making them easily misdiagnosed by general practitioners (GPs) [8], resulting in delayed diagnosis. The main cause of untimely detection of OSCC is that there are no vital clinical indications that can aid specialists in accurate diagnosis. Various signs may indicate the presence of oral cancer, such as the location, size, color, and appearance of the lesion in the mouth, and a patient’s history of smoking and drinking can provide insight into a potential oral cancer diagnosis. Biopsy is the most commonly used pathological examination method for the detection and diagnosis of oral cancer. Pathologists can diagnose OSCC by observing strips of cells/tissues extracted from the tumor area. Currently, the most accurate diagnosis of cancer is based on the microscopic inspection of biopsy slides. In this diagnostic technique, a specialist removes a tiny piece of the tumor and prepares slides stained with hematoxylin and eosin (H&E) for microscopic examination. However, such a diagnosis is a difficult process that requires specialized knowledge and a large amount of time and can be easily misleading. Therefore, early diagnosis of OSCC is essential for effective treatment, which can increase patients’ chances of survival while also reducing their risk of morbidity and mortality. The earlier stage of oral cancer has a 5 to 6-year survival rate of approximately 69.5%; however, in the later stage, this rate decreases to 31.6% [9,10]. The cost of oral cancer treatment is roughly 7.36 times more than that of OPMDs in the last stage and 2.64 times higher in the early stage [11]. Therefore, early prediction could minimize the financial impact on patients with oral cancer. Currently, doctors utilize both traditional and advanced methods for the detection of cancer using microscopic images. Due to the high incidence and massive density of laboratories, there is a need for an early system for the diagnosis of oral cancer. Furthermore, employing artificial intelligence techniques, particularly machine learning (ML) and deep learning (DL), to improve diagnosis provides promising outcomes. CNN-based models are among the finest DL methods for generalizing learning processes on diverse datasets. To train the DL algorithms, the dataset was divided into training, testing, and validation datasets. The optimization process of the CNN model involves learning the robust features of each disease and evaluating the test image to generate results based on the learned features. The models were then capable of recognizing new cases after training. The accuracy of these models may be affected by several factors, including the presence of noisy data in the dataset, scarce and imbalanced datasets, architecture, and the hyperparameters of the models. This study aimed to address the challenges faced by CNN models while identifying histological images to achieve better results, which are essential for the early prediction of OSCC. To achieve the goal of this study, the images in the dataset were improved to eliminate noise and deal with problems that take a long time and require expensive computers. This was performed using a combination of deep and machine learning methods. In this study, various techniques were utilized to diagnose OSCC using histopathological images. In addition, the diagnosis of OSCC was accomplished by fusing features from deep learning models with features of color, texture, and shape derived from the conventional GLCM, HOG, and LBP algorithms.

The main contributions of our work are as follows:

- Enhance histological images of oral cancer using two distinct filters for improved image quality and feature extraction.
- Fine-tune hyperparameters of convolutional neural network (CNN) models to optimize classification performance and enhance model accuracy.
- Oral cancer cell histology images can be effectively diagnosed with a hybrid technique utilizing CNN models and the SVM algorithm.
- The PCA algorithm was utilized to reduce the number of features in the high-dimensional OSCC dataset.
- The diagnosis of the histological images of OSCC cells using the SVM algorithm, which is based on the hybrid features extracted by the CNN model; it combines these characteristics with the color, texture, and shape data extracted using the GLCM, HOG, and LBP algorithms.

The rest of this article is structured as follows: In Section 2, we present a detailed review of relevant recent studies conducted. Section 3 discusses the material and techniques utilized for analyzing and interpreting histopathological images in the context of OSCC diagnostics. The evaluation results of the proposed methods are outlined in Section 5. Section 6 offers a comprehensive discussion and comparison of the proposed techniques with the other existing models. Finally, the conclusion is drawn in Section 7.

## 2. Related Work

This section aims to shed light on the trends and problems associated with OSCC diagnosis. This study demonstrates how several researchers have used various approaches to attain promising diagnostic accuracy. In particular, we offer a rigorous investigation to detect, evaluate, and summarize the facts regarding oral cancer diagnosis, prevention, prognosis, and treatment. Many studies have been carried out in recent years that combine histopathological images and white light images that is, images captured with smartphones, speech, and genomic data, using machine learning for various applications fields [12,13]. In 1996, researchers initially applied the findings of a machine-generated neural network toy to healthy, precancerous, and cancerous oral smears [14]. The SVM is a commonly used algorithm in the pathology of oral cancer, accounting for 42.10% of all applications; artificial neural networks (ANNs) have a proportion of 24.47; logistic regression (LR) has a proportion of 21.16; and CNN-based applications have a proportion of 12.37 [15]. The field of ANNs has expanded significantly, which is an important development. This expansion has been driven by the rise in data accessibility, dedicated advanced computing architecture for the execution of machine learning algorithms, and the use of Graphics Processing Units (GPUs) that help in quick data processing [16]. The author [17] presents a transfer learning strategy where they used AlexNet to extract deep features from OSCC biopsy images with a classification accuracy of 90.06%. The authors in [18] used a deep learning model to diagnose OSCC from histopathology images. They used three different CNN models, that is, VGG16, Resnet50, and InceptionV3, and then concatenated them to extract and classify deep features. Their algorithm attained accuracies of 89.16%, 90.83%, 94.16%, and 96.6%, respectively. Authors in [19] discussed how to diagnose oral cancer from the microscopic images of biopsy specimens. The area containing the lesion was segmented by hand, and then, the morphological and structural features were extracted for further examination. These features were then assigned to five different machine-learning classification algorithms. This study [20] also described a technique for diagnoses based on various features such as texture, shape, and color by analyzing the images. Furthermore, these features were then input to the SVM, LR, and decision tree (DT) classifiers. SVMs outperformed the other techniques for categorizing color and texture features with better accuracy.

The Mobile-Net model [21] was used for identifying OSCC histopathological examinations performed on tissue samples of 20 patients, which were first scanned using an ex vivo confocal microscope immediately after surgical resection. The sensitivity and specificity of these models were 47% and 96%, respectively. The study conducted by [22] offered four deep-learning-based approaches for classifying and separating oral cancer lesions. Their techniques produced adequate pixel segmentation outcomes for the lesions. A spectroscopic technique based on replications and auto-imaging was described in [23] for the detection of OSCC at the boundaries of malignancy in 104 patients. The area under the curve (AUC) showed that the system had a success rate of 82% with a 3 mm safety margin. For the histopathological imaging of oral cancer, [24] proposed a two-step technique for the early detection and segmentation of epithelial tissues and stroma. In this study, they tested four different models: Xception, ResNet50, ResNet101, and MobileNetv2, and the best accuracy was achieved by the combined use of Xception and SWT. The segmentation of OSCC in the pharynx and cavity was demonstrated by [25] using CNNs for the segmentation of OSCC in the pharynx and cavity. For the diagnosis, 34 and 45 oral and pharyngeal lesion video clips, respectively, were analyzed. The oropharyngeal lesions had 111 and 117 frames taken from the video, respectively. Tumor segmentation was performed using three FCNN models. ResNet outperformed the other models, with a dice coefficient of 65.48% and 76.02%. Authors in [26] proposed a technique using a CNN, which identified 673 images of oral dysplasia in the epithelial layer from 53 patients. Data augmentation was performed on the images to address overfitting. A novel CNN method along with texture features was proposed by [27] for the detection of oral cancer. Their methodology is composed of two interrelated components: oral cancer detection, marking of the region of interest (ROI), and semantic segmentation. A wavelet-transform-based method yielded detection results with a sensitivity and specificity of up to 96.76% and 71.18%, respectively. For the identical problem of oral cancer detection, [28] proposed a deep neural-based adaptive fuzzy system that exploited machine learning for classification and achieved an accuracy of 93%. This study [29] used ResNet to perform binary classification on images of oral pathology; they were able to reach an accuracy of 91.13%. The author [30] presented an early technique for the segmentation and classification of histological images that has recently been performed using CNN-based image processing algorithms with sensitivity and specificity of 86% and 89%, respectively. Authors in [31] classified images of dysplastic tissue into four categories using transfer learning, attaining accuracies of 94% and 90% during training and testing, respectively. The authors [32] identified 73 potential patients, of which 22 were found to have benign conditions and 51 had malignancy. There was an early equal distribution of male and female patients (37 female and 36 male patients). According to the findings of a 10-fold cross-validation analysis, the average specificity and sensitivity of ANNs for predicting oral cancer were 85.71% and 60.00%, respectively. The overall accuracy of ANNs was 78.95%. In the study presented in [33], 34 prognostic factors were taken from a database to create four machine learning models: LR, DT, SVM, and K-Nearest Neighbor (KNN). Their aim was to use these models to predict disease progression. The decision tree model showed the highest success in identifying disease progression with an accuracy of 70.59%. This study [34] performed, two computer vision techniques based on deep learning were tested for early and timely detection and categorization of oral lesions for oral cancer. One approach was image classification using ResNet-101, and the other was object detection using a Faster R-CNN. The prior method showed an F1 score of 87.07% for identifying images with lesions and 78.30% for identifying images that required a referral. In contrast, the object detection approach had an F1 score of 41.18% for detecting lesions requiring referral.

## 3. Materials and Methods

**Figure 1:**
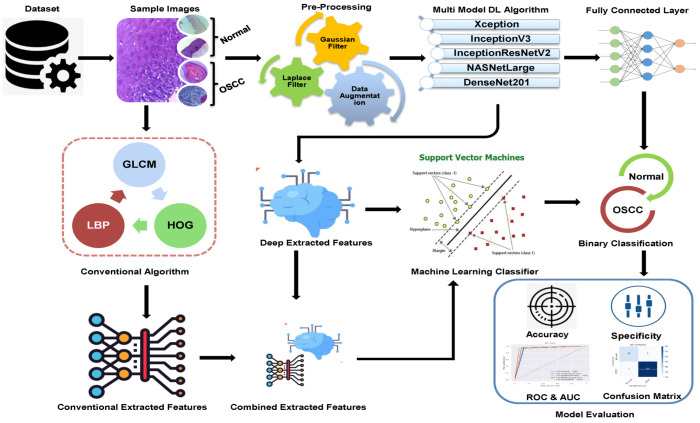
Proposed Methodology of OSCC.

The methodologies and materials used in the histological images for the early detection of OSCC are summarized in this section and are shown in Figure 1. The initial step in this study was to improve the quality of all histological images in the OSCC dataset because they contained artifacts. Three strategies were implemented using five distinct models to achieve the objectives of this study. First, we used deep learning models to classify the dataset using Xception, InceptionV3, InceptionResnetV2, NASNetLarge, and DenseNet201 models. The second method involved using a hybrid approach that blends deep learning algorithms and an SVM to classify images. The third strategy in this study is to classify the dataset using SVM, which employs hybrid features extracted from the combination of DL with the HOG, GLCM, and LBP algorithms.

### 3.1. Dataset Description

**Figure 2:**
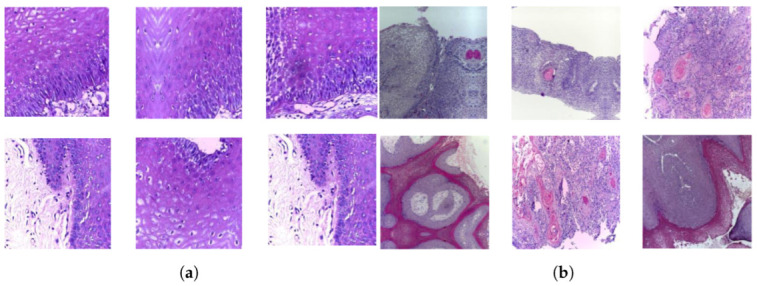
Samples of histopathological images (**a**) Normal and (**b**) OSCC samples.

A publicly accessible collection of OSCC histopathological image data was used to evaluate the systems presented in this study [35]. Biopsy slides were collected from two reputed healthcare service institutions: Ayursundra Healthcare Pvt. Ltd. and Dr. B. Borooah Cancer Institute. This oral cancer dataset consists of two binary classes: normal and OSCC. Images were captured using a Leica DM 750 microscope with camera model ICC50 HD, at 100× (10× objective lens 10× eyepiece) magnifications (size 2048 × 1536 pixels). The dataset contained 5192 images captured from biopsy slides at a magnification of 100×. The images in this data collection were obtained by biopsy under local anesthesia. A pathologist made the diagnosis based on these biopsies. Out of 5192 histopathological images, 2494 (48%) were classified as normal and 2698 (52%) as malignant cases of OSCC. This study focused on these images for analysis. A sample of the normal and OSCC images included in the dataset is shown in Figure 2.

### 3.2. Pre-Processing of Histological Images

A vital step in biomedical image processing is preprocessing, which ensures the images are correctly set up for analysis to achieve high accuracy. The use of CNN models for image analysis requires substantial computing power, and the input images must be correctly formatted for the model to function effectively. Biopsy slides may vary in color because of dark areas, bloodstains, or other medicinal solutions. To standardize the color of the images, the average RGB color was calculated for every image to adjust their scale and ensure color consistency throughout the dataset. In the last step of preprocessing, the images were cleaned of artifacts, the contrast was increased, and the edges of the region of interest (ROI) were revealed using Gaussian and Laplacian filters [36]. These filters are commonly used in image processing to sharpen or enhance the edges in an image, which can be useful for highlighting the ROI in the images. The edges of the image were highlighted using Gaussian and Laplacian filters. Following preprocessing, a Gaussian noise filter was applied to the input images, which suppressed high-frequency information while preserving low-frequency details. This aids noise reduction and image averaging. The Gaussian noise filter is a low-frequency linear filter that is particularly effective in removing blur and noise from the images. The smoothing parameter of the Gaussian filter can be adjusted to manage the degree of smoothing applied to an image. Equation (1) illustrates the operation of a Gaussian noise filter.

(1) $$h\left(x\right)=\frac{1}{\sigma \sqrt{2\pi }}e\frac{-{\left(x-\mu \right)}^{2}}{2\sigma^{2}}$$

The standard deviation of a random variable is denoted by σ, whereas μ indicates the mean of *x*. The images were then processed using a Laplacian filter to highlight the edges of the lesions in the pathological tissue images, as expressed in Equation (2).

(2) $$\nabla^{2}f=\frac{df}{d^{2}x}+\frac{d^{2}f}{d^{2}y}$$

*x* and *y* denote the pixel’s location in the images.

### 3.3. Deep Learning Model

DL is considered an essential technology in the field of artificial intelligence, and its techniques have been widely adopted across various industries. DL requires large amounts of data to effectively train the models. The more data there are, the better the model performs on the unobserved data. However, collecting and labeling large amounts of data can be time consuming and expensive. Furthermore, the quality of data is essential because inaccurate or skewed data can affect the results. The performance of the system depends on the size, complexity, and amount of available data, and training a DL model can take a few hours, several days, or even weeks. Despite the data and computational requirements, deep learning can achieve advanced performance on a wide range of tasks. For example, DL models have been able to achieve near-human performance in tasks such as image classification, object detection, and natural language understanding. However, it is important to note that the performance of deep learning models can vary depending on the specific task and quality of the data. Additionally, DL can be vulnerable to overfitting, where the model becomes too specialized for the training data and performs poorly on unseen data; therefore, it is essential to validate the model with such data and fine-tune the model to avoid overfitting. In general, DL is a powerful and effective method that may yield outstanding outcomes for a wide range of tasks, but it requires large amounts of data and computing ability.

Among AI systems, CNN models are exceptional in their ability to extract deep feature maps. To ensure that CNN models can make accurate classifications when tested, data collection is required as part of their training. Various types of features are extracted at different levels and layers of a deep neural network during the feature extraction process. For example, the first layer is responsible for extracting color characteristics, the second layer is accountable for obtaining feature engineering, the third layer is liable for extracting texture characteristics, etc. [37]. Additionally, convolutional neural network models have a number of layers, each of which is optimized for different purposes. A CNN comprises several layers, starting with a convolutional layer and moving on to the pooling layers, auxiliary layers, and a fully connected layer. These three layers are briefly described in the following paragraphs.

#### 3.3.1. Convolution Layer

The convolution layer is a fundamental component of CNN. It is responsible for extracting features from the input data, such as images or audio. Three key parameters control the functionality of a convolutional layer: *p*-step, zero padding, and filter size [38]. The filter size defines the pixels *f*(*t*) in the filter that extract information from the input image *x*(*t*) through a sliding window. Zero padding was applied to maintain the original image size. The *p*-step controls the movement of the filter over the input image; where a *p*-step of 1 indicates that the filter moves by 1 pixel at all, a *p*-step of 2 indicates that the filter moves by 2 pixels at a time. Equation (3) shows the filtering procedure.

(3) z(t)=(x∗f)(t)=∫x(a)f(t−a)da

where f(t) shows the filter, x(t), and z(t) represent the input and output image, respectively.

#### 3.3.2. Pooling Layer

The large number of parameters generated by the convolutional layers may lead to a significant computational burden for CNNs. To address this issue, pooling layers were introduced into the CNN [39,40]. These layers decreased the spatial size of the output feature maps from the convolutional layers. The two methods used to reduce image dimensions are average pooling and max pooling. Max pooling involves using the filter size to choose a cluster of image pixels, identifying the one with the highest value, and replacing all the selected pixels with that one value by using Equation (4). The second method for reducing image dimensions is average pooling. This process chooses a group of pixels in the image using a filter size, calculates the average value of these pixels, and replaces them with a single value that signifies the average. This can be seen in Equation (5)

(4) $$z\left(m;n\right)=\max_{i,j}=1\ldots kS\left[\left(m-1\right)p+i;\left(n-1\right)p+i\right]$$

(5) $$z\left(m;n\right)=\frac{1}{k^{2}}\sum_{i,j=1\ldots k}S\left[\left(m-1\right)p+i;\left(n-1\right)p+j\right]$$

where *S* represents the number of filter pixels; *i* and *j* are the dimensions of the input image; *k* represents the image size, and *p* denotes the step size.

#### 3.3.3. Fully Connected Layer

A dense layer, often known as a fully connected layer (FCL), is a fundamental building block in neural network architectures. Its main function is to convert the bidirectional representation of the input data into a unidirectional representation. In a fully connected layer, each neuron receives input from all the neurons in the preceding layer and applies a set of learned weights to the inputs before passing them through an activation function to produce an output. Finally, the sigmoid activation layer assigns a label to an image by evaluating its similarity to available classes. It uses a probability value between 0 and 1 to sort the image into the appropriate class. There are several alternative classes of activation functions available, such as (ReLU), which allows positive values to pass through unchanged, but converts negative values to zero, as described in Equation (6).

(6) $$\mathrm{ReLU}\left(i\right)=\max\left(0,i\right)=\left\{\begin{array}{cc} i, & i\geq 0\\ 0, & i<0 \end{array}\right\}$$

**Figure 3:**
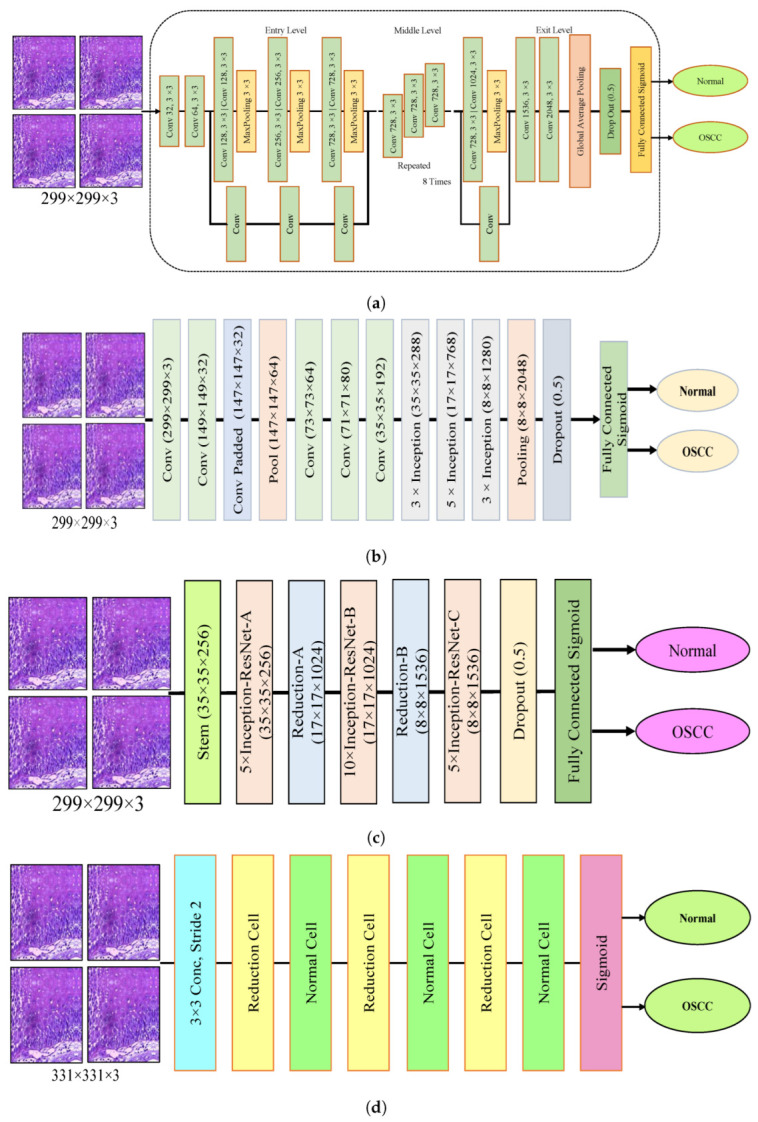

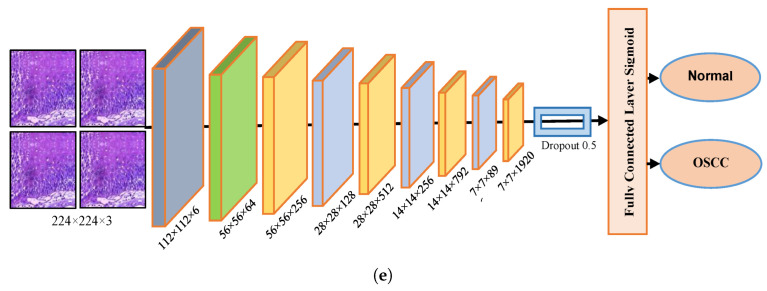
Deep learning architectures (**a**) Xception model (**b**) InceptionV3 model (**c**) InceptionResNetV2 model (**d**) NASNetLarge model, and (**e**) DenseNet201 model.

The dropout layer addresses the overfitting caused by many network parameters by randomly excluding a portion of them during each training iteration. The dropout rate was set to 0.5, meaning that only half of the neuron information was passed during each iteration, which resulted in an increase in the training time for the model. This segment assesses the histopathological images of OSCC with five distinct pre-trained models, namely Xception, InceptionV3, InceptionResNetV2, NASNetLarge, and DenseNet201 models, along with their fundamental architectures, as shown in Figure 3.

### 3.4. Hybridization of CNN with SVM

In this section, we present a new technique that combines a CNN with an SVM. The motivation behind this approach is to use a hybrid solution to tackle the challenges of excessive processing resource usage and slow performance of CNN models. This hybridized approach is intended to overcome these challenges by being computationally efficient, requiring low-cost resources, and providing fast training for the dataset, which results in highly efficient diagnostic outcomes. The hybrid approach comprises two components. The initial step of this hybrid approach is the enhancement of histological images of OSCC, followed by deep feature maps extracted using a variety of CNN models, such as Xception, InceptionV3, InceptionResNetV2, NASNetLarge, and DenseNet201. The extracted deep features are then stored in the feature vector and transferred to the SVM. In CNN models, deep feature maps are used as inputs, and an SVM is used to replace the final layers. SVM provides the final diagnostic results by accurately classifying each vector feature.

#### 3.4.1. Extracting Deep Features

CNN models have a unique feature extraction capability. During the training phase, the CNN extracts deep features that are used to categorize the images during the testing phase. Feature extraction is performed through multiple layers and levels, each extracting unique features [37]. Deep features were extracted from histological images of OSCC using Xception, InceptionV3, InceptionResNetV2, NASNetLarge, and DenseNet201 models; then, they are stored in feature vectors, which are subsequently fed to the machine learning (ML) model to perform the classification. CNNs are known for extracting high-dimensional features, and to address this issue and reduce the complexity of the dataset, Principal Component Analysis (PCA) was applied to extract essential feature maps, thus reducing the dimensionality of the data.

#### 3.4.2. Support Vector Machine (SVM)

The SVM model was utilized to replace the final layers of the CNN model. The SVM utilizes the features extracted from the Xception, InceptionV3, InceptionResNetV2, NASNetLarge, and DenseNet201 models and predicts with high accuracy and a faster training speed. The SVM represents all values of the dataset in n-dimensional space, where n denotes the number of features present in the dataset [41]. The algorithm represents each feature value in a dataset with absolute coordinates. Subsequently, it aims to create multiple lines of separation among the values of different classes known as hyperplanes. The model then selects the optimal hyperplane that amplifies the gap between classes. The margin is the region between the hyperplane and the closest samples from each class, called the support vectors. There are two types of SVM algorithms: linear and nonlinear. One may choose between a linear SVM algorithm and a nonlinear algorithm. Linear SVM is implemented when the dataset may be divided along linear dimensions. However, a nonlinear SVM is utilized if the dataset cannot be linearly separated. The most commonly used kernels are linear, sigmoid, polynomial (poly), and radial basis functions (RBFs). The selection of the kernel is contingent on the data and problem at hand. The data were segregated in this study using the RBF and poly kernels.

**Figure 4:**
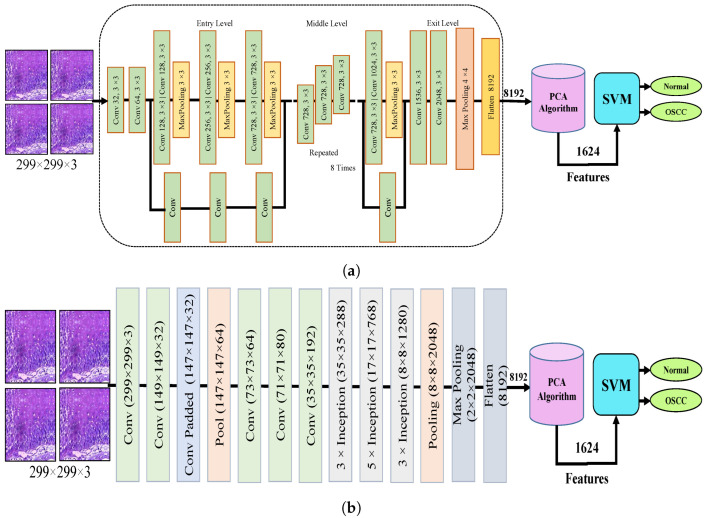

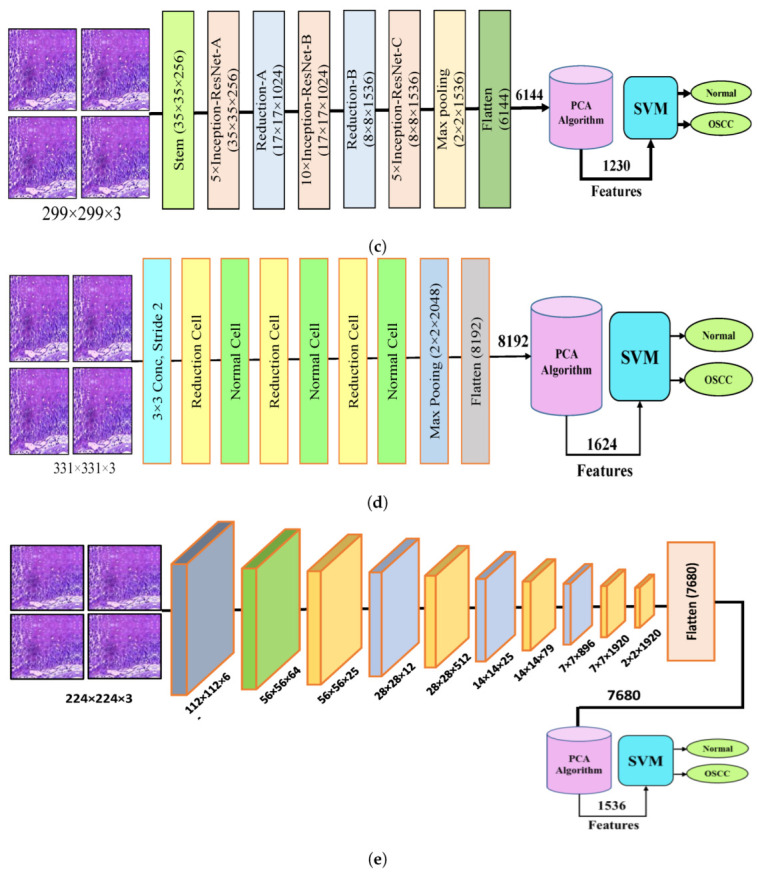
CNN and SVM hybrid models (**a**) Hybrid model between Xception and SVM (**b**) Hybrid model between InceptionV3 and SVM (**c**) Hybrid model between InceptionResNetV3 and SVM (**d**) Hybrid model between NASNetLarge and SVM, and (**e**) Hybrid model between DensNet201 and SVM.

Figure 4 shows the hybrid method employed for the diagnosis of OSCC histological images. In this section, a method for diagnosing histological images of oral cancer using an SVM is described. This method is based on deep feature extraction utilizing the Xception, InceptionV3, InceptionResNetV2, NASNetLarge, and DenseNet201 models. The methodology outlined in this section consists of the following phases: The first phase involved preprocessing the histopathological images by removing noise and increasing the contrast of the region of interest. The optimized images were fed to the Xception, InceptionV3, InceptionResNetV2, NASNetLarge, and DenseNet201 models. In the second step, features were extracted through different models and stored as vector features with a size of (n × m), where n is the number of samples and m represents the number of feature vectors. The number of samples n in the proposed dataset is 5192, where the number of feature vectors m for Xception, InceptionV3, and NASNetLarge is 5192 × 8192. For InceptionResNetV2, the number of feature vectors was 5192 × 6144, and for DenseNet201, it was 5192 × 7680.

It should be noted that the dataset was massive and contained high-dimensional features for each histopathological image. To address this issue, we employed PCA, which assists in compressing the data while preserving the essential properties of the feature vectors. By utilizing PCA, the number of feature vectors was decreased by 20%, which equates to 5192 × 1624 for Xception, InceptionV2, and NASNetLarge, 5192 × 1230 for InceptionResNetV2, and 5192 × 1536 for DenseNet201. Finally, these low-dimensional features were used for classification by the SVM algorithm to determine whether the image was normal or OSCC, as shown in Figure 4.

### 3.5. SVM-Based Hybrid CNN Deep Features with GLCM, HOG, and LBP

The proposed approach involves the extraction of hybrid features using multiple CNN models, specifically Xception, InceptionV3, InceptionResNetV2, NASNetLarge, and DenseNet201. These features were fused with those obtained from traditional algorithms, specifically HOG, GLCM, and LBP. The resulting hybrid features are fed to an SVM for classification to attain a high level of accuracy in the diagnostic process. In this section, we describe our proposed work, which includes the following steps. The first step involves preprocessing the histopathological images by removing noise and increasing the contrast of the region of interest. The optimized images were fed to the Xception, InceptionV3, InceptionResNetV2, NASNetLarge, and DenseNet201 models. In the second step, features were extracted through different models and stored as vector features, as anticipated in the preceding section. By utilizing PCA, the number of feature vectors was decreased by 20%, which equates to 5192 × 1624 for Xception, InceptionV2, and NASNetLarge, 5192 × 1230 for InceptionResNetV2, and 5192 × 1536 for DenseNet201.

In the third step, three hybrid algorithms GLCM, HOG, and LBP were used after the enhancement of the histopathological images to extract the most important features, including shape, color, and texture, to achieve high classification accuracy. LBP is a texture descriptor that is used in computer vision and image processing. This method is based on comparing the intensity of each pixel to that of its neighboring pixels. The LBP descriptor captures the spatial relationship between pixels and provides details regarding the texture of an image. The LBP descriptor was calculated by comparing the intensity of the central pixel to that of its neighboring pixels. If the intensity of the central pixel is greater than the intensity of its neighboring pixel, the corresponding bit in the LBP code is set to 1; otherwise, it is set to 0. This process is repeated for all the surrounding pixels, resulting in a binary code that represents the texture of the image. The target pixel is replaced by 48 neighboring pixels using Equation (7) for a 7 × 7 patch. The LBP algorithm compares the gray-level intensity of the target pixel $\left(g_{c}\right)$ with the gray-level intensity of its neighboring pixels $\left(g_{p}\right)$.

(7) $$LBP_{R,P}=\sum_{p=0}^{p-1}s\left(g_{p}-g_{c}\right)2^{p}$$

where *R* represents the distance from the central pixel to its neighboring pixels, $\left(g_{p}\right)$ is the gray-level intensity of the neighboring pixels, $\left(g_{c}\right)$ is the gray-level intensity of the central pixel, and *P* denotes the number of neighboring pixels considered in the calculation.

The LBP algorithm can differentiate pixels by evaluating the density of the image and comparing the intensity of each pixel with that of its neighboring pixels, thereby producing 203 texture features. GLCM is a technique used to extract textural features from an image. The image was initially converted to grayscale to build the GLCM. The GLCM is calculated by counting the instances of each pair of gray levels co-occurring in the image for a specific offset or distance between the two pixels. The offset is defined by the user in any direction. The GLCM method utilizes spatial information to compute statistical texture characteristics by assessing the connection between pairs of pixels according to their direction θ and distance *d*, which together characterize the position of each pixel relative to the others. The GLCM algorithm evaluates the relationship between each pixel and its neighboring pixels by considering four different directions θ:0${}^{\circ }$, 45${}^{\circ }$, 90${}^{\circ }$, and 135${}^{\circ }$. θ determines the distance between pixels *d*, and is controlled by the direction θ between the pixels. The *d* is equal to 1, when θ is either 0${}^{\circ }$ or 90${}^{\circ }$, and *d* = $\sqrt{2}$, when θ is either 45${}^{\circ }$ or 135${}^{\circ }$. The GLCM technique generated 13 statistical texture features as a result of this process.

The HOG is a widely used feature representation in computer vision and image processing. Generating a histogram of gradient orientations in specific localized areas of an image makes it possible to obtain the shape and texture of the objects within an image. The image was divided into small regions, and within each region, a histogram of gradient orientations was computed. This feature descriptor is robust to changes in lighting and viewpoint and is widely used for object detection and recognition. The descriptor is generated by computing the gradient orientations for each pixel in the image, and then quantizing them into a fixed number of bins to form an HOG for each region. These histograms are then concatenated to form the final feature descriptor. The proposed method employs a larger number of histogram bins in different image regions. The input images were converted to grayscale after resizing to 64 × 128 pixels. Equation (8) was applied to obtain the gradient for each pixel, and 231 features were extracted using the HOG.

(8) $$\left\{\begin{array}{c} dx=P(x+1,y)-p(x,y)\\ dy=p(x,y+1)-p(x,y) \end{array}\right.$$

**Figure 5:**
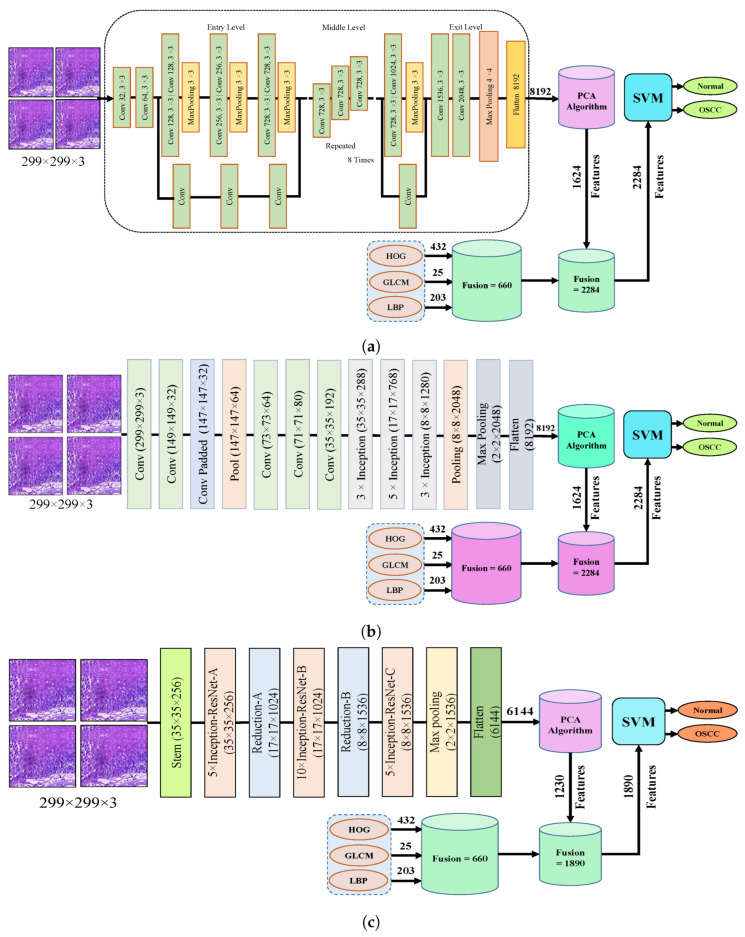

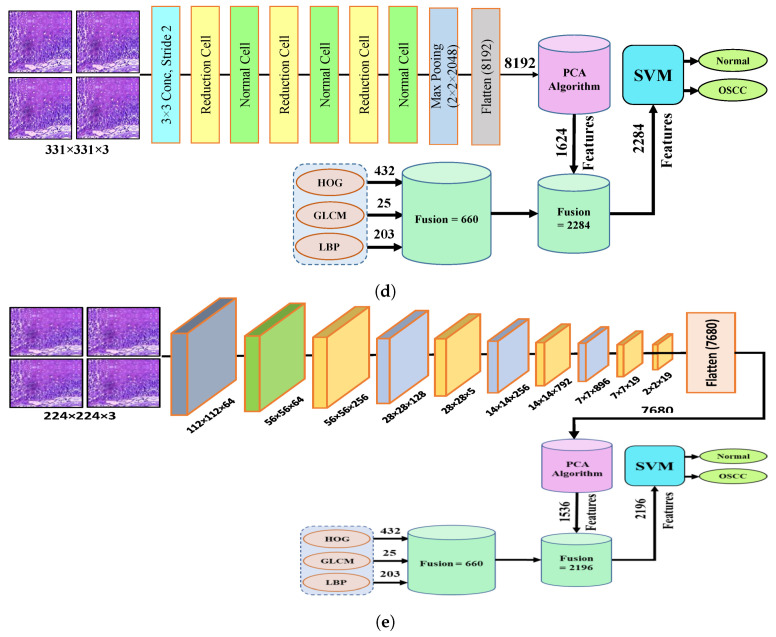
Hybrid features between CNN model, with HOG, GLCM, and LBP (**a**) Hybrid Features between Xception with HOG, GLCM, and LBP (**b**) Hybrid features between InceptionV3 with HOG, GLCM, and LBP (**c**) Hybrid features between InceptionResNetV2 with HOG, GLCM, and LBP (**d**) Hybrid features between NASNetLarge with HOG, GLCM, and LBP, and (**e**) Hybrid features between NASNetLarge with HOG, GLCM, and LBP.

The fourth step of the process involves the concatenation of all features extracted from the CNN, such as Xception, InceptionV3, InceptionResNetV2, NASNetLarge, and DenseNet201, with the features obtained through the GLCM, HOG, and LBP. After this merging operation, the size of the dataset increased to 5192 × 2284 from 5192 × 1624 for Xception, InceptionV3, and NASNetLarge; 5192 × 1890 from 5192 × 1230 for InceptionResNetV2; and 5192 × 2196 from 5192 × 1536 for DenseNet201. In addition, these features were fed into the SVM for the classification of the image as normal or OSCC, as shown in Figure 5.

## 4. Evaluation Tools

### 4.1. Confusion Matrics

To evaluate the proposed models, the confusion matrices from each system were used to compute the accuracy, precision, sensitivity, specificity, and AUC using Equations (9)–(13), respectively: True Positive (*TP*) and True Negative (*TN*) are metrics in the confusion matrix that represent the number of correctly identified histological images. False negatives (*FN*) and false positives (*FP*) represent the number of misclassified images.

(9) $$\;\mathrm{Accuracy}\;=\frac{TN+TP}{TN+TP+FN+FP}\times 100\%$$

(10) $$\;\mathrm{Precision}\;=\frac{TP}{TP+FP}\times 100\%$$

(11) $$\;\mathrm{Sensitivity}\;=\frac{TP}{TP+FN}\times 100\%$$

(12) $$\;\mathrm{Specificity}\;=\frac{TN}{TN+FP}\times 100\%$$

(13) $$\;\mathrm{AUC}\;=\frac{\;\mathrm{Sensitivity}\;}{\;\mathrm{Specificity}\;}$$

*TP* represents the number of images that have been truly classified as malignant and *TN* represents the number of images that have been truly classified as normal. *FP* represents the number of normal images that have been incorrectly classified as malignant and *FN* represents the number of malignant images that have been incorrectly classified as normal.

The performance of the hybrid systems was evaluated using a confusion matrix, which consolidated all the samples in the dataset and distinguished between the correctly and incorrectly classified samples, resulting in the calculation of the accuracy and diagnostic accuracy for each class. This table is used to define the performance of a classification algorithm, where the true values are defined along the main diagonal, and the false values are found elsewhere. It typically has two rows and two columns: one for true positives, one for false positives, one for true negatives, and one for false negatives. The entries in the confusion matrix represent the number of times each case was predicted by the model. This is used to evaluate how well the model can predict the true class of an instance.

### 4.2. Receiver Operating Characteristic (ROC)

The ROC is a graphical method for evaluating the performance of binary class classification systems. As the threshold is adjusted for the classification, the ROC curve plots the relationship between the True Positive Rate (TPR) and False Positive Rate (FPR). This tool is widely used for machine learning and data analysis. The TPR is calculated by dividing the number of correctly predicted positive cases by the total number of actual positive cases, whereas the FPR is the number of false positive predictions divided by the total number of actual negative cases. The ROC curve is a useful tool for evaluating the trade-off between the specificity and sensitivity of a classifier and for comparing different classifiers.

### 4.3. Area under the Curve (AUC)

The AUC is a graphical representation utilized to assess the performance of binary classification systems. It displays the relationship between the true positive rate and false positive rate by plotting the true positive rate, which represents the proportion of actual positive observations that are correctly identified as positive, against the false positive rate, which represents the proportion of actual negative observations that are inaccurately classified as positive. The area under the ROC curve (AUC) assesses the classifier’s capability to differentiate between negative and positive classes, with a higher AUC indicating a better classifier performance. It ranges from 0 to 1, where a value of 1 represents a perfect classifier and a value of 0.5 represents a random classifier. An AUC of 0.7 or higher is considered a good classifier in most cases.

### 4.4. Training and Validation Accuracy/Loss

We performed a comprehensive analysis of the model performance throughout the training and validation phases. The graphs show the learning process of the model, with the X-axis showing the number of iterations or epochs and the Y-axis showing metrics such as loss and accuracy. The model gradually improves as it absorbs the data displayed by the training curve, whereas its ability to generalize to new data is assessed by the validation curve. By comparing the training and validation loss and accuracy, this study assessed the potential issues of overfitting or underfitting. The goal was to achieve convergence in the curves, indicating effective learning without overfitting. To prevent overfitting, early stopping was considered if the validation loss started to increase, while the training loss continued to decrease. To successfully diagnose OSCC earlyally, this study aimed to make well-informed decisions about model tuning and enhancements through a thorough analysis and interpretation of the training and validation graphs. This strategy aids in the creation of a reliable and accurate model for histopathological image analysis, thereby improving OSCC diagnosis and treatment.

### 4.5. Data Augmentation

The proposed system was evaluated on the OSCC dataset, which comprised two classes. The dataset was split into normal histopathology classes, which accounted for 48% of the data, and malignant tumor histological images, which constituted the remaining 52%. To obtain optimal results and avoid overfitting, the CNN required a large dataset during the training phase. In the OSCC dataset, there were insufficient images for training the proposed models. Data augmentation was used to expand the histological image dataset for training to address overfitting issues. Data augmentation employs various techniques such as rotation at different angles, shifting, and flipping. Datasets are shown as pre- and post-training data. After the data were increased, it was found that each image in the normal and OSCC classes was augmented by a factor of five. Table 1 displays the number of images in each class after applying the data augmentation step.

## 5. Results

### 5.1. Analysis and Insights Results of Deep Learning Models

The results of the pre-trained models Xception, InceptionV3, InceptionResNetV2, NASNetLarge, and DenseNet201 are presented in this section. These methods utilized pre-trained models from the ImageNet dataset, which contains over a million images spanning more than 1000 classes. However, as OSCC images were not included in the ImageNet dataset, the transfer learning technique was applied to modify the pre-trained models for this specific task. Transfer learning enables CNN models to utilize training data from the OSCC dataset for the classification of new datasets. To achieve high performance, CNN models require a substantial amount of data, which is not always readily accessible, particularly for medical image datasets. Therefore, during the training phase, CNN models may exhibit overfitting. Therefore, data augmentation can be used to mitigate this issue.

All models were trained using the *Adam* optimizer with a *sigmoid* activation function. The batch size for training was set to 32, and a learning rate (α) of 0.0001 was used. The models were trained in a *Kaggle* environment.

The Xception, InceptionV3, InceptionResNetV2, NASNetLarge, and DenseNet201 models yielded excellent outcomes when analyzing the histopathological images for the diagnosis of OSCC. Table 2 provides a summary of the results obtained by the proposed systems. It can be seen that all the models performed well in examining the histopathological images, which can assist physicians and specialists in the rapid diagnosis of OSCC conditions. This is essential because this is a sensitive condition and needs to be detected properly to be treated effectively and quickly. The results showed that the NASNetLarge model demonstrated a high level of performance with an accuracy of 94.44%, specificity of 95.80%, precision of 90.32%, sensitivity of 87.52%, and F1 score of 88.88%. DenseNet201 has a high precision of 96.77%, specificity of 98.87%, and AUC of 94.70%.

**Figure 6:**
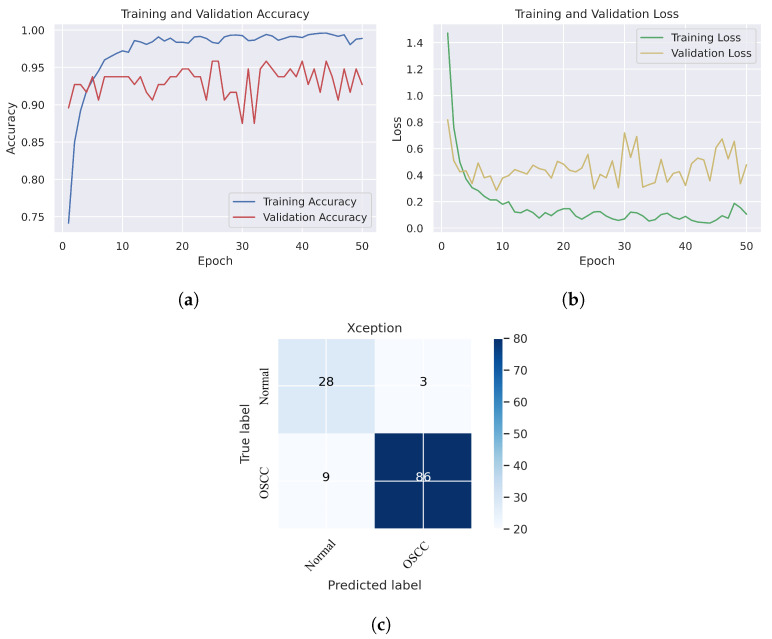
Xception model. (**a**) Training and validation accuracy (**b**) Training and validation loss (**c**) Confusion matrix.

**Figure 7:**
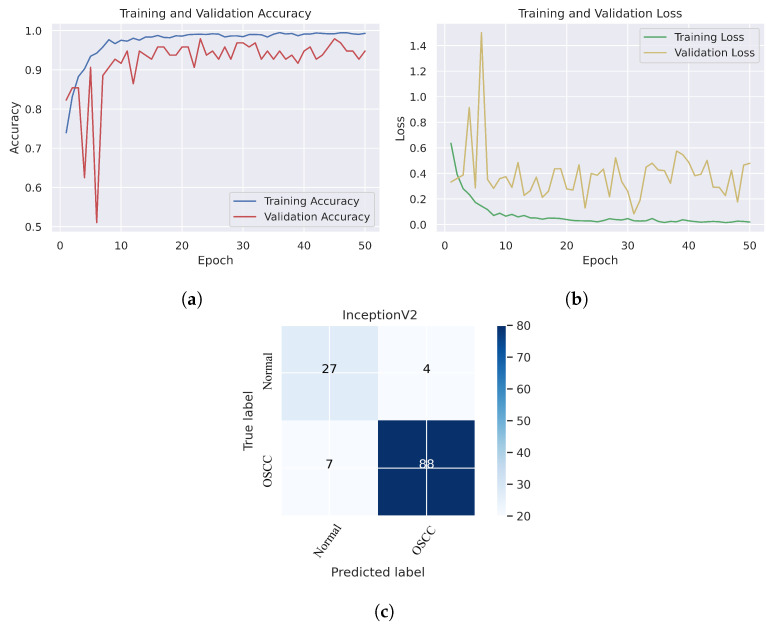
InceptionV2. (**a**) Training and validation accuracy (**b**) Training and validation loss (**c**) Confusion matrix.

In Figure 6, we can see that the Xception model performed well in assessing histopathological images for OSCC diagnosis, attaining an overall accuracy of 90.47% and 90.52% for diagnosing cancer images. Furthermore, it correctly classified the normal images with 90.32% accuracy. Figure 6c shows that several histopathological images had incorrect labels whereas, nine OSCC class images were incorrectly classified as normal, However, three normal class images were incorrectly identified as OSCC images. Figure 7 illustrates the performance of the InceptionV3 model in assessing histopathological images for the diagnosis of OSCC, achieving an overall accuracy of 91.26% and 92.63% for diagnosing cancer images. In addition, 87.09% of the normal images were accurately detected by the system.

**Figure 8:**
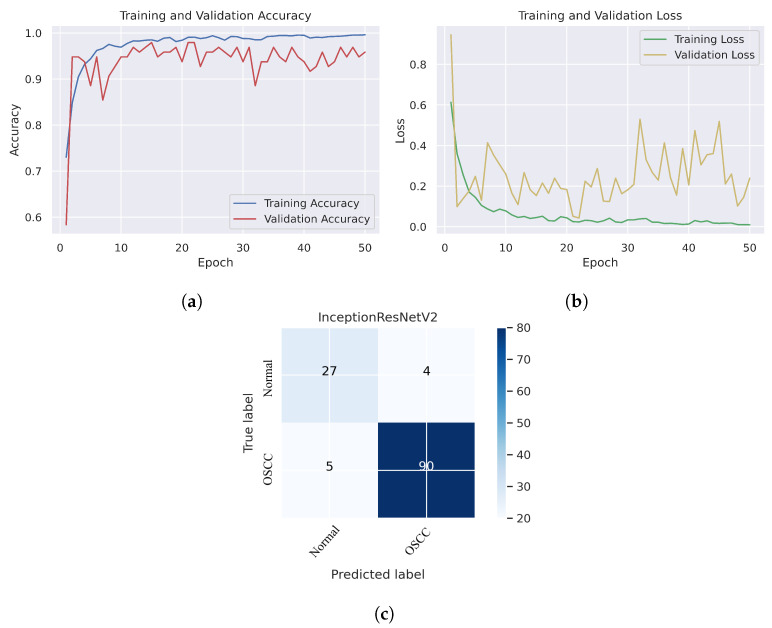
InceptionResNetV2. (**a**) Training and validation accuracy (**b**) Training and validation loss (**c**) Confusion matrix.

**Figure 9:**
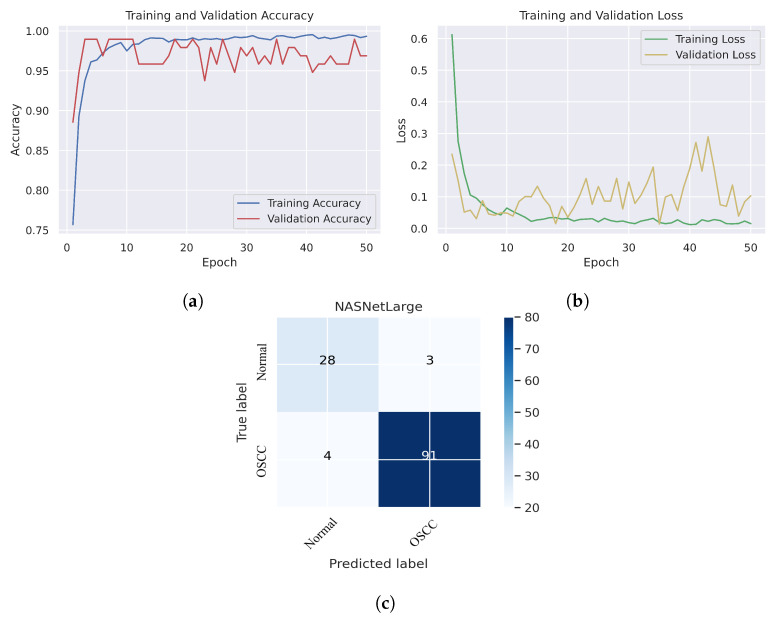
NASNetLarge. (**a**) Training and validation accuracy (**b**) Training and validation loss (**c**) Confusion matrix.

**Figure 10:**
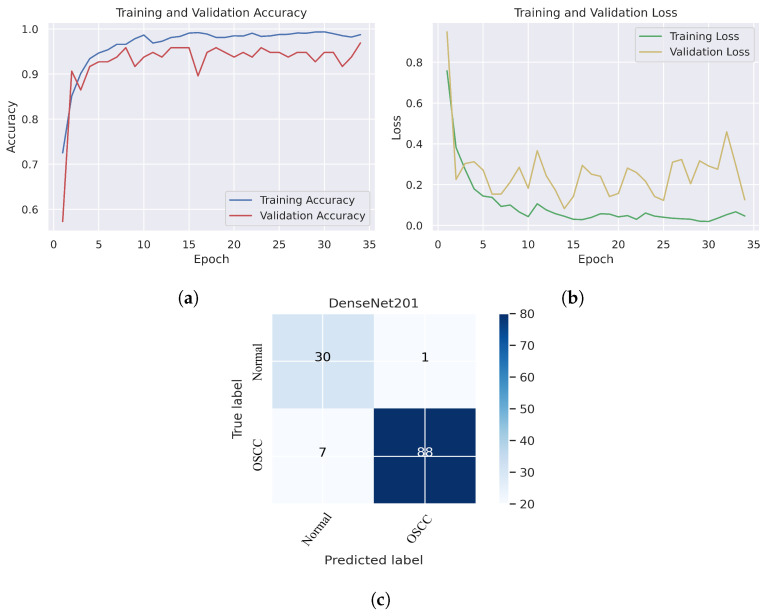
DenseNet201. (**a**) Training and validation accuracy (**b**) Training and validation loss (**c**) Confusion matrix.

As shown in Figure 7c, several histopathological images were incorrectly labeled. Seven OSCC images were identified as normal; however, four normal images from the dataset were classified as OSCC. Figure 8 depicts the performance of the InceptionResNetV2 model, obtaining an overall accuracy of 92.85% and 94.73% for diagnosing cancer images. Furthermore, this model classifies normal images with an accuracy of 87.09%. It can be observed in the confusion matrix shown in Figure 8c that few histopathological images were misclassified. Five OSCC images from the dataset were marked as normal; however, four images from the normal class were identified as OSCC. This model has fewer incorrect classifications than the Xception and InceptionResNetV2 models. The visualized performance analysis of the NASNetLarge model can be seen in Figure 9. Figure 9c presents the confusion matrix of this model, where very few images were incorrectly classified. The overall accuracy of the NASNetLarge model was reported to be 94.44% and 95.78% for diagnosing cancer images. The NASNetLarge model provided an accuracy of 90.32% for the correct classification of normal images. From the confusion matrix, we can see that numerous histopathological images were labeled incorrectly, among which four images of the OSCC condition were labeled as normal and three images from the normal class were identified as OSCC. The performance evaluation of DenseNet201 is shown in Figure 10. This model achieved an overall accuracy of 93.65% and 92.63% for diagnosing cancer images. In addition, it accurately detected normal images with an accuracy rate of 96.77%.

Figure 10c shows that several images were classified incorrectly when the DenseNet201 model was employed. We noted seven such images from the OSCC class that were labeled as normal, and one image from the normal class was labeled as OSCC. In terms of accuracy and generalization, NASNetLarge outperformed the other models, that is, Xception, InceptionV3, InceptionResNetV2, and DenseNet201, as can be seen in the training and validation graphs. NASNetLarge demonstrated higher accuracy for both the training and validation datasets, depicting accurate learning and prediction of OSCC. Furthermore, as shown in Figure 9a, the validation accuracy for NASNetLarge is closely aligned with the training accuracy, indicating a minimum risk of overfitting. In terms of the loss function, NASNetLarge demonstrated the lowest training and validation loss values with respect to the other models, indicating higher convergence and learning power, as illustrated in Figure 9b. Conversely, some of the other models exhibited larger gaps between training and validation losses, resulting in potential overfitting. The convergence of the accuracy and loss curves in NASNetLarge further underscores its effectiveness in learning from the data. These findings support the conclusion that NASNetLarge yielded better results than the other models, making it a promising approach for the diagnosis of OSCC using deep learning.

### 5.2. Performance Evaluation of the Combined CNN–SVM Method: Experimental Results

**Figure 11:**
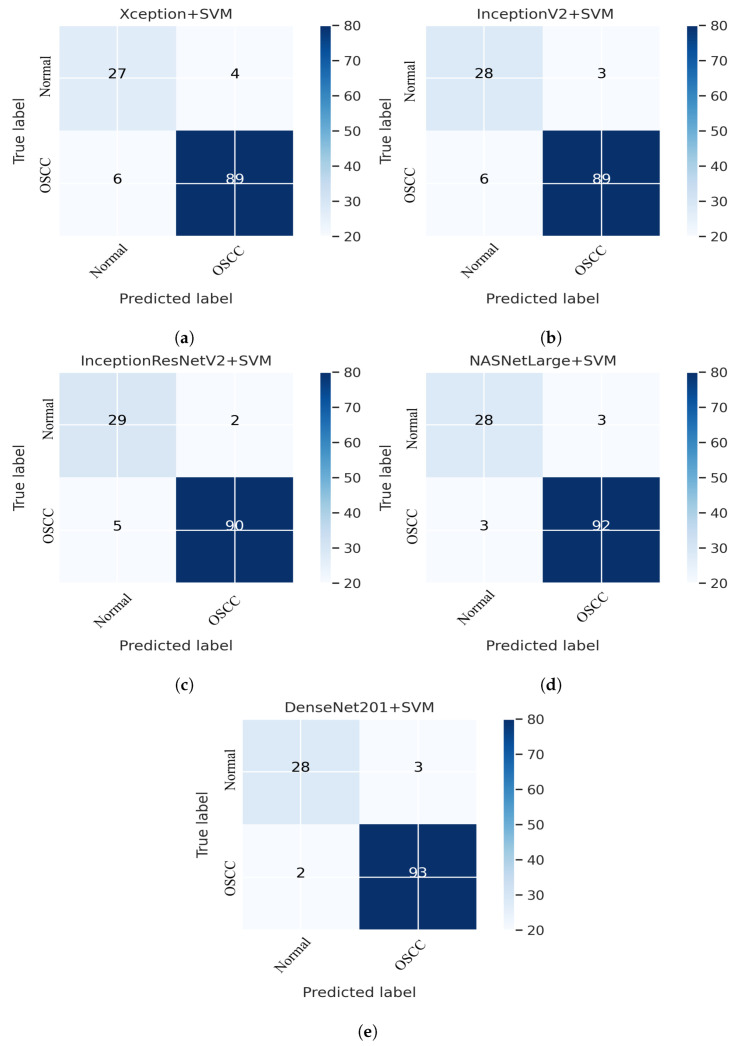
CNN and SVM hybrid models confusion matrices. (**a**) Xception + SVM (**b**) InceptionV3 + SVM (**c**) InceptionResNetV2 + SVM (**d**) NASNetLarge + SVM and (**e**) DenseNet201 + SVM.

This section addresses the performance of the hybrid technique for histopathological image diagnosis using CNN models and the SVM algorithm for rapid and efficient prediction of OSCC conditions. In this hybrid approach, the CNN initially extracts deep features using various models. These extracted deep features are then stored as vector features and passed to the SVM algorithm for classification. In this study, we employed five different CNN models with an SVM: Xception + SVM, InceptionV3 + SVM, InceptionResNetV2 + SVM, NASNetLarge + SVM, and DenseNet201 + SVM. The results of the hybrid technique performed on the OSCC dataset are listed in Table 3. Figure 11 shows the overall performance of the hybrid system using the confusion matrix. Based on these findings, DenseNet201 performed better than the other hybrid approaches in terms of accuracy, sensitivity, F1 score, and AUC. Figure 11e shows that the DenseNet201 + SVM model performed well in assessing histopathological images for the quick diagnosis of OSCC, achieving a total accuracy of 96.03% and an accuracy of 97.89% for OSCC Images. It identifies normal images with 90.32% accuracy. Furthermore, two OSCC class images were incorrectly classified as normal and three normal class images were incorrectly classified as OSCC.

### 5.3. Performance Evaluation of Hybrid Deep Feature Model with GLCM, HOG, and LBP

This section presents an overview of the performance of the SVM for diagnosing OSCC using histopathological images, which is based on a combination of deep features extracted from CNN and the traditional algorithms HOG, GLCM, and LBP. This method uses a hybrid approach to extract features by combining deep learning and traditional algorithms from histopathological images for the classification of OSCC. First, the deep learning models Xception, InceptionV3, NASNetLarge, InceptionResNetV2, and DenseNet201 were used to extract the deep features from the images, which were then reduced in dimensionality using the PCA algorithm. Next, traditional algorithms such as GLCM, HOG, and LBP were used to extract an additional 660 features per image. The resulting dataset has a feature vector size of 5192 × 2284 for Xception, InceptionV3, and NASNetLarge; 5192 × 1890 for InceptionResNetV2, and 5192 × 2196 for DenseNet201. Finally, an SVM was used to classify the images as normal or OSCC using a combination of these deep features and traditional algorithm features. Several assessment tools were utilized to evaluate the performance of the proposed system and are discussed in Section 4. This method combines five distinct CNN models with traditional algorithms GLCM, HOG, and LBP; then, it is fed to an SVM for classification. The models used were Xception + (GLCM, HOG, and LBP) + SVM, InceptionV3 + (GLCM, HOG, and LBP) + SVM, InceptionResNetV2 + (GLCM, HOG, and LBP) + SVM, NASNetLarge + (GLCM, HOG, and LBP) + SVM, and DenseNet201 + (GLCM, HOG, and LBP) + SVM. The results of this hybrid technique of the OSCC dataset are shown in Table 4. It should also be noticed that in comparison to several other methods, DenseNet201 performs noticeably better. DenseNet201 + GLCM, HOG, and LBP fed to SVM attained 97.00% accuracy, 96.77% precision, 90.90% sensitivity, 98.92% specificity, and 93.74% F1 score.

**Figure 12:**
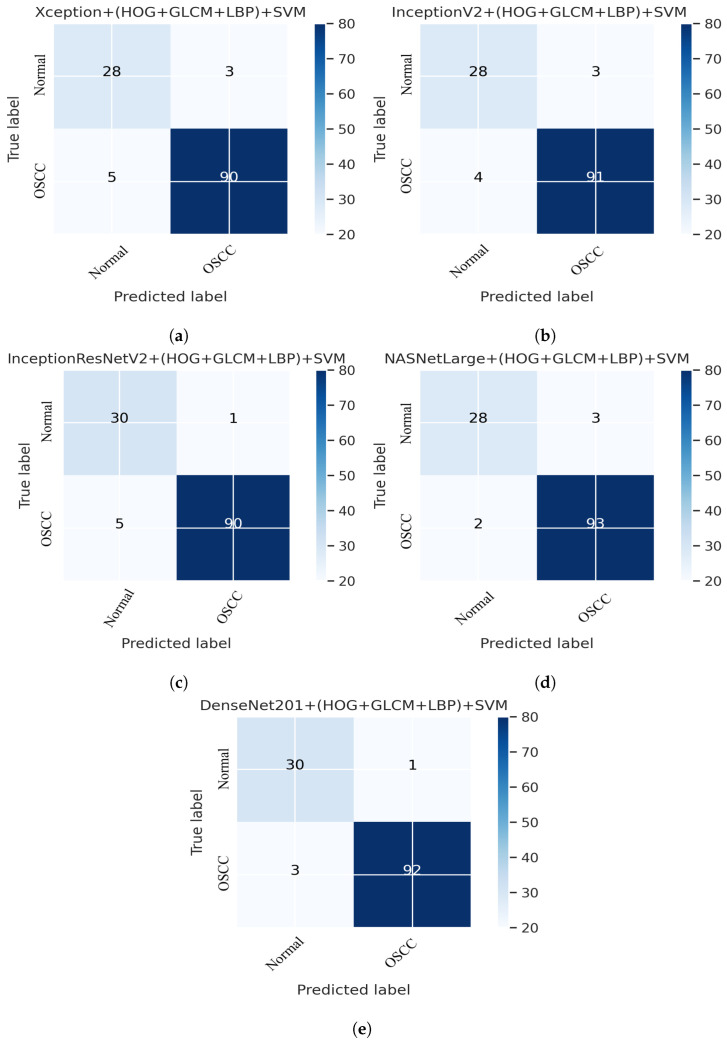
Deep Feature with (GLCM, HOG, and LBP) confusion matrices. (**a**) Xception + (GLCM, HOG, and LBP) + SVM (**b**) InceptionV3 + (GLCM, HOG, and LBP) + SVM (**c**) InceptionResNetV2 + (GLCM, HOG, and LBP) + SVM (**d**) NASNetLarge + (GLCM, HOG, and LBP) + SVM (**e**) DenseNet201 + (GLCM, HOG, and LBP) + SVM.

Figure 12 shows the overall performance of the hybrid deep feature model with GLCM, HOG, and LBP of Xception, InceptionV3, InceptionResNetV2, NASNetLarge, and DenseNet201 using confusion matrices. Extensive simulation results show that DenseNet201 performs better than the other models. DenseNet201 + (GLCM, HOG, and LBP) + SVM attained 96.82% accuracy, 96.77% precision, 90.90% sensitivity, 98.92% specificity, and a 93.74% F1 score. DenseNet201+(GLCM+HOG, and LBP) + SVM model performed well in assessing histopathological images for the quick diagnosis of OSCC, achieving a total of 96.82% accuracy, and 96.77% accuracy for the diagnosis of OSCC, respectively. It identifies normal images with 96.84% accuracy. Furthermore, three OSCC class images were incorrectly classified as normal, and one normal class image was incorrectly classified as OSCC, as illustrated in Figure 12e.

### 5.4. Receiver Operating Characteristic (ROC) and AUC

**Figure 13:**
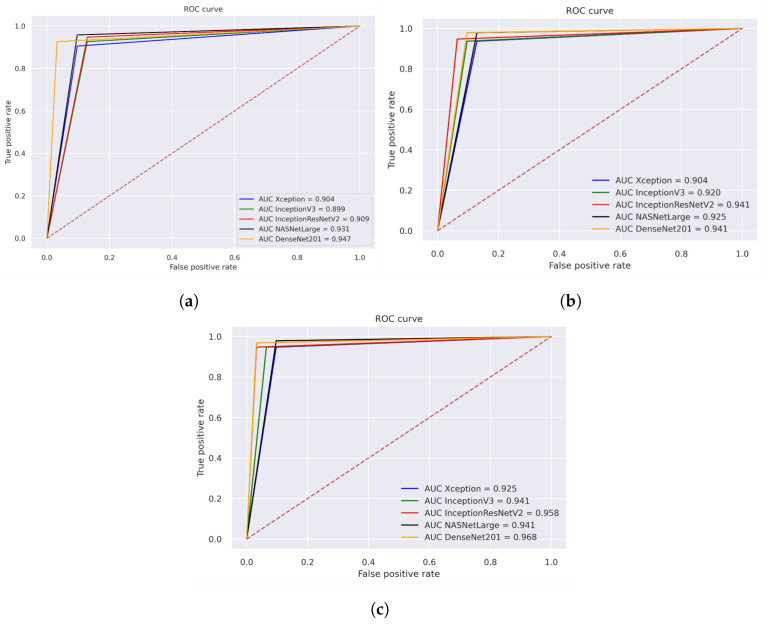
ROC of histopathological images of OSCC (**a**) Deep learning models (**b**) CNN with SVM (**c**) Hybrid Deep Feature with GLCM, HOG, and LBP.

Figure 13 illustrates the performance evaluation of the AUC for the three different strategies for histopathological images. The performance evaluation of Xception, InceptionV3, InceptionResNetV2, NASNetLarge, and DenseNET201 is shown in Figure 13a, achieving overall AUC of 90.4%, 89.9%, 90.90%, 93.10%, and 94.70%, respectively. Figure 13b illustrates the hybrid features of Xception + SVM, InceptionV3 + SVM, and InceptionResNetV2 + SVM, NASNetLarge + SVM, DenseNet201+ SVM, which achieved an overall AUC of 90.40%, 92.00%, 94.10%, 92.50%, and 94.10%, respectively. Figure 13c displays the results of using a fusion of hybrid features including Xception + (GLCM, HOG, and LBP) + SVM, InceptionV3 + (GLCM, HOG, and LBP) + SVM, and InceptionResNetV2 + (GLCM, HOG, and LBP) + SVM, NASNetLarge + (GLCM, HOG, and LBP) + SVM, DenseNet201 + (GLCM, HOG, and LBP) + SVM, which achieved an overall AUC of 92.50%, 94.10%, 95.80%, 94.10%, and 96.80%, respectively.

## 6. Discussion of Proposed Methods

In the proposed method, the use of hybrid techniques was investigated to develop an automated and precise early diagnosis of OSCC. We proposed three different strategies to achieve this goal, each of which makes use of five different models that combine these techniques in various ways to extract features from the images and classify patterns. Three distinct filters were used to preprocess each image in the OSCC dataset. Moreover, to obtain optimal results, the CNN algorithm requires a large dataset during the training phase. Consequently, the dataset did not have sufficient images to train the model. A data augmentation technique was applied to increase the number of histological images in the dataset during the training phase to address overfitting problems. This study aimed to assist clinicians in improving the early detection of OSCC, increasing the accuracy of diagnosis, assisting in the diagnostic process, reducing workload, and being cost-effective. This study aimed to improve the early prediction of OSCC, which can lead to better diagnostic results. The proposed model seeks to increase the accuracy of OSCC diagnosis and reduce computational time by combining various models and methodologies, which can help clinicians make more informed decisions about patient treatment. This is an automated method that can assist doctors by providing a second opinion of images, reducing the time required for manual analysis, and reducing the workload of doctors and pathologists. This is achieved by utilizing a combination of CNNs, SVM, and other feature extraction techniques, such as GLCM, HOG, and LBP.

One of our key contributions to this study is the application of five different models for comprehensive comparison. By using a diverse range of models, we were able to thoroughly evaluate the performance of transfer learning and make informed comparisons. This approach not only provides a comprehensive understanding of the strengths and weaknesses of each model but also allows us to identify the most effective approach for the problem at hand. In addition to other state-of-the-art CNN models, this contribution represents a significant step forward in many potential fields and highlights the importance of comparing multiple models when evaluating new techniques. The five models utilized in this work are the Xception, Inceptionv3, InceptionResNetV2, NASNetLarge, and DenseNet201 models, which achieved overall accuracies of 90.47%, 91.26%, 92.85%, 94.44%, and 93.65%, respectively. The second strategy is the extraction of hybrid features using both CNN and SVM, which is the key contribution of this study. The features are extracted using different models and stored in a feature vector. It should be noted that the dataset was massive and contained high-dimensional features for each histopathological image. To address this issue, we employed PCA, which assists in compressing data while preserving the essential properties of the feature vectors. Then, we fed these features to the SVM as inputs for better classification.

This method addresses several issues associated with the CNN models. Training a CNN model requires a large amount of computational power and can be time-consuming. Consequently, this approach is easy to implement and can efficiently train a dataset on a low-end computing device. To address these challenges, researchers have developed techniques to increase the efficiency and cost-effectiveness of the training process. In the implementation of the proposed model, five distinct models were utilized: Xception + SVM, Inceptionv3 + SVM, InceptionResNetV2 + SVM, NASNetLarge + SVM, and DenseNet201 + SVM, which attained an overall accuracy of 92.06%, 92.85%, 94.44%, 95.23%, and 96.03%, respectively. The third procedure is a hybrid feature fusion of the CNN with GLCM, HOG, and LBP, which is the key contribution of the proposed study. In this procedure, the features were extracted using different models, stored in a feature vector, and passed through PCA for dimensionality reduction. These features are then fed into the SVM for better classification. For the implementation of this strategy, five distinct models were utilized: Xception + (GLCM, HOG, and LBP) + SVM, Inceptionv3 + (GLCM, HOG, and LBP) + SVM, InceptionResNetV2 + (GLCM, HOG, and LBP) + SVM, NASNetLarge + (GLCM, HOG, and LBP) + SVM, and DenseNet201 + (GLCM, HOG, and LBP) + SVM, which attained an overall accuracy of 93.65%,94.44%,95.23%,96.03%, and 97.00%, respectively. The overall accuracies of the proposed models for histopathological image diagnosis are listed in Table 5. The results indicated that the CNN model combined with GLCM, HOG, and LBP achieved the best overall accuracy among the other models. The highest accuracy in identifying histopathological images was achieved using an SVM classifier that coupled the features of DenseNet201 with GLCM, HOG, and LBP. This technique has an overall accuracy of 97.00%.

**Figure 14:**
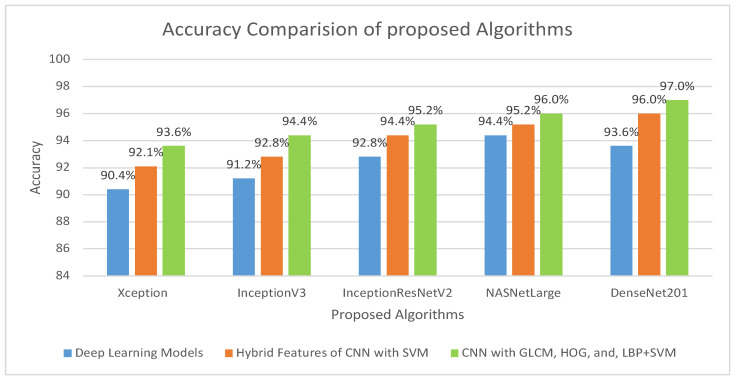
Comparison of various models in terms of accuracy.

The overall accuracy of the proposed methodology for histopathological images of OSCC is shown in Figure 14. It is evident that for the diagnosis of OSCC, DenseNet201 mixed with GLCM, HOG, and LBP together with SVM performed better than the other techniques.

**Figure 15:**
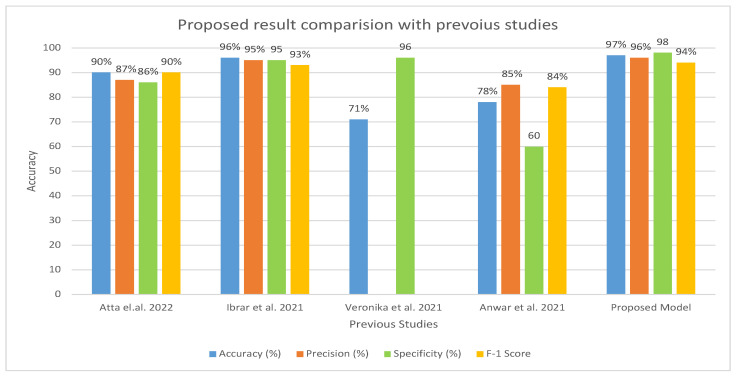
Comparison of Proposed Model with the existing model [17,18,21,32] in terms of accuracy, precision, specificity, and F1 score.

Table 6 and Figure 15 present the evaluation of the proposed model in comparison to earlier research. A comparison of our method with previous research shows that it is more efficient and effective. In contrast to earlier studies that were restricted to a few indicators, our system was assessed using a wider range of evaluation measures. Earlier investigations reported an accuracy range of 71–96%, whereas our approach exhibited an accuracy of 97%. Furthermore, the specificity of our system was recorded as 98.92% compared to earlier studies, which had a specificity ranging from 60.01% to 95.01%. Similarly, the precision of our system was measured as 96.77%, which outperformed the precision reported in previous studies, ranging from 85.71% to 95.16%.

## 7. Conclusions

The model proposed in this study provides an early diagnosis of OSCC using histopathological images. This study aimed to assist clinicians by improving the early detection of OSCC, increasing the accuracy of diagnosis, helping in the diagnostic process, reducing workload, and being cost-effective. This study employed three strategies, each using five models. The first strategy, transfer learning, achieved an overall accuracy ranging from 90.47 to 94.44%. The second strategy, a hybrid of CNN and SVMs, had an overall accuracy range of 92.06–96.03%. The third strategy, a hybrid of CNN, GLCM, HOG, and LBP, results in an overall accuracy range of 93.65–97.00%. The reason for the highest accuracy achieved by the proposed model is the use of customized layer and preprocessing techniques on the dataset. Second, among the three different strategies, the third strategy, which combines a CNN with features extracted using GLCM, HOG, and LBP, and then classifies the features using SVM, gave better results because of the combination of various feature extraction techniques. Each of these techniques provides a distinct perspective on image data, and their combined use has the potential to provide a more comprehensive understanding of the data, which leads to improved performance. The CNN is good at learning spatial relationships between pixels, the GLCM provides texture information, the HOG characterizes the shape of the objects, and the LBP acquires the local pattern information. A more robust and accurate model is produced by combining these techniques, which enables a more detailed comprehension of the images. The proposed model demonstrates significant improvements in accuracy and efficiency for OSCC diagnosis, enabling more precise and timely treatment decisions.

## Figures and Tables

**Table 1:** Data augmentation of histopathological Images.

Training Phase		
**Classes **	**Before Augmentation**	**After Augmentation**
Normal	2436	12,180
OSCC	2511	12,555

**Table 2:** Ev ological images via deep learning models.

Measures	Xception	InceptionV3	InceptionResNetV2	NASNetLarge	DenseNet201
Accuracy (%)	90.47	91.26	92.85	**94.44**	93.65
Precision (%)	90.32	87.09	87.09	90.32	**96.77**
Sensitivity (%)	75.67	79.41	84.37	**87.52**	81.01
Specificity (%)	96.62	95.65	95.74	95.80	**98.87**
F1 Score (%)	82.34	83.07	85.70	**88.88**	88.23
AUC (%)	89.4	89.90	90.90	93.10	**94.70**

**Table 3:** Evaluation of experimental results for OSCC histopathological images via hybrid CNN–SVM Method.

Measures	Xception	InceptionV3	InceptionResNetV2	NASNetLarge	DenseNet201
	**+ SVM**	**+ SVM**	**+ SVM**	**+ SVM**	**+ SVM**
Accuracy (%)	92.06	92.85	94.44	95.23	**96.03**
Precision (%)	87.09	90.32	**93.54**	90.32	90.32
Sensitivity (%)	81.81	82.35	85.29	90.32	**93.33**
Specificity (%)	95.69	96.73	**97.82**	96.84	96.87
F1 Score (%)	84.36	86.15	89.22	90.32	**91.80**
AUC (%)	90.40	92.00	**94.10**	92.50	**94.10**

**Table 4:** Assessment of SVM performance with Fusion features for early diagnosis of OSCC.

Measures	Xception	InceptionV3	InceptionResNetV2	NASNetLarge	DenseNet201
	**GLCM, HOG**	**GLCM, HOG**	**GLCM, HOG**	**GLCM, HOG**	**GLCM, HOG**
	**&, LBP, SVM**	**&, LBP, SVM**	**&, LBP, SVM**	**&, LBP, SVM**	**&, LBP, SVM**
Accuracy (%)	93.65	94.44	95.23	96.03	**97.00**
Precision (%)	90.32	90.32	**96.77**	90.32	**96.77**
Sensitivity (%)	84.84	87.53	85.71	**93.33**	90.90
Specificity (%)	96.77	96.80	98.90	96.87	**98.92**
F1 score (%)	87.49	88.88	90.90	91.80	**93.74**
AUC (%)	92.50	94.10	95.80	94.10	**96.80**

**Table 5:** The overall result of the proposed methodology.

Strategies	Models	Accuracy(%)
Deep Learning Model	Xception	90.47
	InceptionV3	91.26
	InceptionResNetV2	92.85
	NASNetLarge	94.44
	DenseNet201	93.65
Hybrid Features of CNN with SVM	Xception + SVM	92.06
	InceptionV3 + SVM	92.85
	InceptionResNetV2 + SVM	94.44
	NASNetLarge + SVM	95.23
	DenseNet201 + SVM	96.03
Hybrid Features Fusion of CNN with GLCM, HOG, and, LBP + SVM	Xception + GLCM, HOG, LBP + SVM	93.65
	InceptionV3 + GLCM, HOG, LBP + SVM	94.44
	InceptionResNetV2 + GLCM, HOG, LBP + SVM	95.23
	NASNetLarge + GLCM, HOG, LBP + SVM	96.03
	DenseNet201 + GLCM, HOG, LBP + SVM	97.00

**Table 6:** Overall result comparison with previous studies.

Existing Techniques	Accuracy(%)	Precision(%)	Specificity (%)	F-1 Score (%)	Models
Atta el.al [17]	90.00	87.69	87.38	90.15	AlexNet
Ibrar et al. [18]	96.00	95.16	95.01	93.71	Concatenation
Veronika et al. [21]	71.21	-	96.00	-	Mobile Net
Anwar et al. [32]	78.95	85.71	60.01	84.68	ANN
Proposed Model	97.00	96.77	98.92	93.74	DenseNet201

## Data Availability

Results from this study’s dataset, which can be found at https://www.kaggle.com/datasets/ashenafifasilkebede/dataset (accessed on 21 October 2023) back up the proposed method.

## Abbreviations

The following are some of the abbreviations that can be found in this manuscript:

OSCC	Oral squamous cell carcinoma
PCA	Principal component analysis
CNN	Convolutional neural network
DL	Deep learning
SVM	Support vector machine
LBP	Local Binary Pattern
HOG	Histogram of Oriented Gradients
GLCM	Gray-Level Co-Occurrence Matrix
